# Estimates of Prevalence Rates of Cancer Patients With Children and Well-Being in Affected Children: A Systematic Review on Population-Based Findings

**DOI:** 10.3389/fpsyt.2021.765314

**Published:** 2021-11-25

**Authors:** Laura Inhestern, Johanna Christine Bultmann, Lene Marie Johannsen, Volker Beierlein, Birgit Möller, Georg Romer, Uwe Koch, Corinna Bergelt

**Affiliations:** ^1^Department of Medical Psychology, University Medical Center Hamburg-Eppendorf, Hamburg, Germany; ^2^Department of Social Work, Münster University of Applied Sciences, Münster, Germany; ^3^Department of Child and Adolescent Psychiatry, Psychotherapy and Psychosomatics, University Medical Center Münster, Münster, Germany; ^4^Department of Medical Psychology, University Medicine Greifswald, Greifswald, Germany

**Keywords:** parental cancer, children, cancer, oncology, distress, well-being, prevalence

## Abstract

This review assessed population-based estimate rates of cancer patients with minor and young adult children (≤ 25 years), children and young adults having a parent with cancer as well as the psychosocial situation and well-being of children and young adults affected by parental cancer. Eighteen publications on population-based studies were included. Studies varied in the age ranges of both cancer patients and children. The prevalence rates of cancer patients having children ranged from 14 to 24.7% depending on the sample structure (e.g., age, gender). Studies reported that between 1.6 and 8.4% of children resp. young adult children have a parent with a history of cancer. Seven publications reported on the psychosocial situation or well-being in children and young adults affected by parental cancer. Estimate rates of psychosocial problems, psychiatric diagnoses or distress ranged between 2.5 and 34% of children depending on the method of measurement and outcome. The differences in the sample structure between the studies impeded the comparison of prevalence rates. However, the findings help to determine the need for specific support services and health care planning. The results emphazise the importance to routinely include issues on the parental role of patients and questions on the well-being and coping of children into psychooncological care. If necessary, support should be provided to families living with a cancer diagnosis.

## Introduction

Cancer is a pervasive stressor not only for the patients themselves but also for the whole social circle (1–3). All family members are confronted with the life-threatening diagnosis and may struggle with disruption of their daily lives. Patients with cancer parenting dependent children are emotionally burdened and experience specific stressors due to the double load of being a patient and a parent (4, 5). The needs as a patient can counteract with the parental role leading to feelings of insufficiency and guilt (6, 7).

Children depending on their parents suffer when a parent is diagnosed with cancer and can show elevated levels of distress (8–10). Additionally, they experience cancer specific stressors such as dealing with uncertainty or concerns about the parent (11–13). Moreover, parents' capacities caring about their children emotionally and practically may be reduced due to physical and mental strains (11).

There is a growing awareness that families with dependent children having a parent with cancer are in need for tailored, specialized and systematic professional support while dealing with the destabilization and distress caused by cancer (14–16).

Recommendations and interventions providing support for affected parents and children have been published (14, 15, 17). These mostly focus on providing help in the current situation of the parental disease and preventing long-term difficulties in the children.

Given the growing evidence that parental cancer is a risk factor for psychosocial problems in children and the fact that health care guidelines call for taking families and children of cancer patients systematically into account and provide them with appropriate services (18, 19), estimations of prevalence rates of cancer patients with dependent children, children having a parent with cancer and well-being of affected children are essential to organize the health care system and appropriate support services.

Until now, many studies investigating the impact of parental cancer on parents themselves or their children are based on selected samples e.g., families seeking for psychosocial support, families attending health care services, or families participating in research studies (20–23). Results based on such selective samples are systematically biased. Hence, they may not provide reliable population-based prevalence rates of cancer patients with dependent children or children affected by parental cancer. A large percentage of parents with cancer and their children, who require support, may not have access to specific services and may not use corresponding services or participate in research studies. Representative population samples must be examined in order to obtain unbiased prevalence data of numbers of affected parents and children as well as prevalence data on psychosocial situation and well-being in children. These data may help to determine the need for specific support services and for rigorous health care planning.

Hence, the purpose of this systematic review was to provide an overview of estimate rates on the prevalence of parents with cancer, on children having a parent with cancer and on psychosocial situation in children from population-based studies.

## Methods

We conducted a systematic review using the PRSIMA (Preferred Reporting Items for Systematic Reviews and Meta-Analyses) guidelines (24). For preparation of the review, a protocol was developed to guide the realization of the systematic review regarding the objective and the planned methods. The protocol was not published.

### Search Strategy

We searched the databases CINAHL, Medline, PsycINFO, and PSYNDEX in January 2018 with updates in April 2019, February 2020 and September 2021. We did not limit the search with regard to year of publication. Key search terms included the following terms: (prevalence^*^ OR population-based OR epidemiology OR survey^*^ OR incidence^*^) AND (parental cancer^*^ OR parental illness^*^ OR parental disease^*^ OR parental physical illness^*^ OR parent with cancer OR disabled parent^*^). We also conducted a systematic search of citations and references of included publications to identify further relevant studies.

### Eligibility Criteria

Publications were included if they met the following criteria: accessibility of full text, published in a peer-reviewed journal, written in English or German, including data on children and young adults (≤ 25 years) having a parent with cancer or including data on parental status of patients with a history of cancer, reporting population estimates of parental status of patients with cancer or population estimates of children and young adults (≤ 25 years) having a parent with cancer. Publications were defined as population-based when they were carried out in a sample of the general population e.g., through sampling strategies or when they were based on data from registries (e.g., cancer registry). Publications were excluded if data was only reported for parental illness in general and not for parental cancer separately. Studies on the psychosocial situation and well-being of children and young adults affected by parental cancer were included if they reported any information on psychosocial outcomes (e.g., psychosocial problems, distress/stress levels, quality of life, internalizing or externalizing problems, psychiatric diagnoses).

### Study Selection

**Figure 1:**
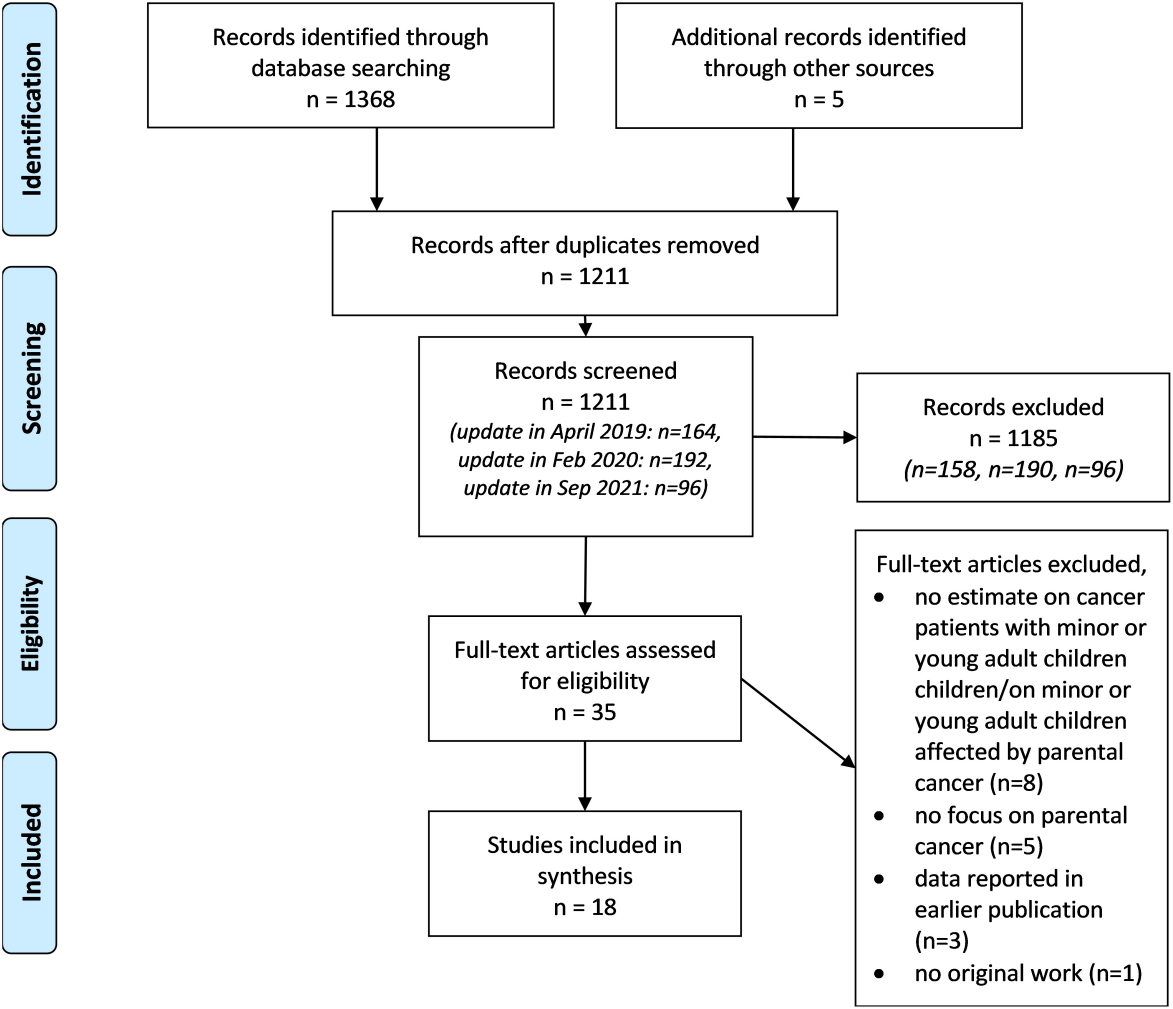
Flow diagram for study selection and reasons for exclusion.

Titles and abstracts of the publications identified were screened according to the predefined inclusion and exclusion criteria (Supplementary Material 1). When title and abstracts were relevant or when eligibility was unclear, the full text was retrieved. Identified full texts were screened based on the inclusion and exclusion criteria. If there was uncertainty about the eligibility after assessing the full text, the eligibility was discussed in the study team. The flow diagram of the selection process is presented in Figure 1.

### Data Extraction and Quality Assessment

Two members of the research team performed data extraction independently using a data extraction form. The standardized form included the following information: citation details, source of information, study population, response rate, estimate/prevalence of parental cancer/affected children and if applicable, ascertainment and information on the psychosocial situation or well-being of children. In some included publications, the estimate of the prevalence or the well-being of children was not the main focus of the study but available from published information in the article.

To assess the quality of the studies two members of the research team independently rated each study using modified criteria adapted from the Newcastle Ottawa Scale (25). Applied criteria comprised rating on the sample selection (incl. representativeness, response rate, sample size, assessment of exposure of cancer) and, if applicable, on the assessment of well-being or the psychosocial situation of children having a parent with cancer (including assessment instrument, data source, statistical tests). All criteria could be rated as “yes,” “no,” “unclear,” or “not applicable” (Supplementary Material 2).

### Analysis Strategy

The data extracted from the included articles were synthesized descriptively and narratively. Since the included age ranges of children and/or young adults and the basic populations differ between studies, we only conducted descriptive synthesis. In case of multiple articles based on the same sample, we described the estimate rates for the article with the most complete study sample or the additional estimate rates separately (e.g., prevalence of parental cancer and information of psychosocial situation/well-being). Due to the heterogeneity of the characteristics of included study samples, we refrained from calculating pooled estimate rates.

## Results

Titles and abstracts of 1.211 articles identified from our systematic literature search were examined (Figure 1). Of these, we retrieved 35 full texts and screened them for eligibility with regard to the predefined inclusion and exclusion criteria. After the full text screening, we excluded 17 articles for several reasons (Figure 1). Three articles were excluded (26–28) since articles on the same study reported data on more complete study samples (29, 30). Hence, in total we included 18 articles in our synthesis.

### Study Characteristics

The 18 articles were based on 14 different samples. Three articles (three samples) reported estimate rates on the prevalence of cancer patients with minor or young adult children (30, 31), whereas 11 articles reported estimate rates on children or young adults having a parent with cancer (29, 32–41). Two articles reported both, estimate rates of cancer patients with minor or young adult children and children and young adults having a parent with cancer (42, 43). Three articles reported prevalence of parental cancer and prevalence on psychosocial situation and well-being in children (44–46). Since they were based on a study sample which is covered by other included articles, these three articles were only included with regard to the prevalence of psychosocial situation in children.

Most articles were from western countries (Table 1). Since most of the studies were solely based on registry data, the quality of the study was acceptable to high for the majority of included publications (Supplementary Material 2).

**Table 1:** Study characteristics and estimates of parents affected with cancer from *n* = 5 articles.

**References**	**Country**	**Database and data source**	**Total sample (*N*)**	**Response rate**	**Estimate on parents with cancer and children**	**Characteristics of parents with cancer: mean age; gender; main diagnoses**	**Characteristics of children: number; age; gender**
Ernst et al. (30)	Germany	Cancer registries, Parental report	*N* = 8.144 cancer survivors (exclusion of highly lethal cancers: digestive and lower respiratory organs, brain and other parts of CNS), age at diagnosis: 25–55 years	41.4%	53.7% (*n* = 1.809) of 3.370 respondents had at least one child ≤ 21 years at time of the diagnosis (22.4% of 8.144 potentially eligible participants)	Mean age: 49 years; gender: 74% female; main diagnoses: > 50% breast cancer	NR^‡^
Inoue et al. (42)	Japan	Cancer registry and medical records	23.185 cancer patients admitted to Hospital (between Jan 2009 and Dec 2013), 8.412 between 20 and 59 years, exclusion of 1.722 (no linkage possible, *in situ* carcinoma), age: 20–59 years	NA^†^	24.7% (*n* = 1.650) of 6.693 cancer patients had minor children	Mean age: 46.6 years (fathers), 43.7 years (mothers); gender: 55.7% female Main diagnoses: gastric, lung, colorectal cancer (fathers), breast, uterus, gastric cancer (mothers)	*n* = 2.593 children age: 0–17 years, M = 11.2 years gender: NR^‡^
Syse et al. (43)	Norway	National population registry and cancer registry	84.202 women with cancer, 67.554 men with cancer, age: 17–70 years	NA^†^	13.5% (*n* = 11.374) women with cancer had minor children, 14.0% (*n* = 9.469) men with cancer had minor children; 15.9% (*n* = 13.450) women with young adult children, 15.6% (*n* = 10.568) men with young adult children	Mean age: NR^‡^; gender: 55.3% female; main diagnoses: testicular, prostate, colorectal cancer (fathers), breast, uterine/cervical cancer (mothers)	*n* = 34.677 (<18 years); *n* = 31.592 (19–25 years) age: minors 0–18 years (33/23% not yet born at diagnosis), young adults 19–25 years (7% not yet born at diagnosis) gender: NR^‡^
Weaver et al. (31)	USA	Representative sample of the civilian non-institutionalized population, Parental report	*N* = 17.173 cancer survivors, *n* = 13.385 excluding exclusively non-melanoma or unknown skin cancers (*n* = 3.788)	67.8–74.3%	14% (*n* = 1.769) cancer survivors had minor children; 18.3% (*n* = 341) recently diagnosed (<2 years earlier) cancer survivors had minor children	Mean age: NR^‡^; gender: 79% female; main diagnoses: breast, or uterine/cervical cancer, and melanoma	*n* = 3.193; age at diagnosis: 33.5% not yet born; 30.5% 0–5 years, 27.2% 6–12 years, 8.9% 13–17 years gender: 49.5% female

† *not applicable (register-based data);*

‡ *not reported*.

### Prevalence of Cancer Patients With Minor and Young Adult Children

Overall, five articles reported estimates of the prevalence of cancer patients with minor or young adult children or parents with a history of cancer (Table 1). Among the publications, the age ranges of children and young adults varied (Table 1). The total sample size of cancer patients or cancer survivors included ranged from *N* = 8.144 to *N* = 151.756. Most prevalent diagnoses in cancer patients with minor or young adult children across all studies were breast and uterine/cervical cancer in mothers and cancer of the digestive organs and cancer of the prostate/testis in fathers. In the included articles mothers were more often affected by cancer than fathers.

The estimates of the prevalence of affected parents with children varied between studies. One article refers to a study based on two cancer registries from Germany with cancer survivors (25–55 years at time of the diagnosis, up to 6 years after diagnosis). At time of the diagnosis 22.4% (53.7% of respondents) were parent of at least one child ≤ 21 years (30).

A register-based study from Japan reported a prevalence of 24.7% of cancer patients between 20 and 59 years with minor children (<18 years) (42). In a sample from Norway, 13.5% of 84.202 women with a history of cancer had minor children and 15.9% had young adult children (19–25 years); in 67.554 men with cancer 14% were fathers of minor children and 15.6% of young adult children (19–25 years) (43). Of the minor children 33% (mothers)/23% (fathers) and of the young adult children, 7% had not yet been born at time of diagnosis of the parent (43).

A study from the USA reported a prevalence of 14.0% of cancer survivors with minor children, whereas in 33.5% of the cases, the children were born after diagnosis. In recently (<2 years prior the survey) diagnosed cancer patients, 18.3% had minor children (31).

### Prevalence of Minor and Young Adult Children Having a Parent With Cancer

Thirteen articles reported estimates of children or young adults having a parent with a history of cancer. The specific age ranges of children varied between studies (Table 2). The size of the total sample ranged from *N* = 1.950 to *N* = 3.868.496 children (Table 2).

**Table 2:** Study characteristics and estimates of children and/or young adults having a parent with cancer from *n* = 13 articles.

**References**	**Country**	**Database and data source**	**Total Sample (*N*)**	**Response rate**	**Estimate on children having a parent with cancer**	**Characteristics of parents with cancer: age Gender main diagnoses**	**Characteristics of children: age; gender**
Barkmann et al. (32)	Germany	Representative survey, Parent report	*N* = 1.950 children	73.1%	4.1% (*n* = 79) of the children had a parent with a physical illness, of these 28.8% had a parent with cancer (*n* = 23, 1.2% of the total sample)	NR^‡^	Age: 4–18 years Gender: NR
Benros et al. (33)	Denmark	Civil registration system, psychiatric central register, and cancer registry	*N* = 61.950 children with psychiatric diagnosis in a population-based cohort of *n* = 3.162.109	NA^†^	6.8% (*n* = 4.342) had been exposed to parental cancer, 2.2% (*n* = 1.348) were aged <15 years at the time of the diagnosis of the parent	NR^‡^	Age: <15 years Gender: NR
Chen et al. (29)	Sweden	Nationwide register-based cohort study	*N* = 2.871.242 children	NA^†^	5.5% (*n* = 113.555) children had been exposed to parental cancer	Age: in 75.4% maternal age at child's birth was 20–34 years Gender: NR^‡^ Main diagnoses: NR	Age: 1–18 years Gender: 48.7% female
Chen et al. (34)	Sweden	Register-based data (e.g., multi-generation register, conscription register and cancer registry)	*N* = 465.249 young men undergoing military conscription examination	NA^†^	4.4% (*n* = 20.383) young men had been exposed to parental cancer	Age: in 58.9% paternal age at child's birth was 20–34 years, in 77.8% maternal age at child's birth was 20–34 years Gender: NR^‡^ Main diagnoses: NR^‡^	Age: Approximately. 18 years (0–17 at time of parental diagnoses) Gender: 100% male
Inoue et al. (42)	Japan	Register based data and medical records	2.563 minor children among 1.650 cancer patients; total population of minors in Japan in 2010 22.780.000	NA^†^	0.38% annual incidence of children being exposed to parental cancer in 2010	See Table 1	Age: M = 11.2 years
Jeppesen et al. (35)	Norway	Representative sample and linkage with cancer registry	*N* = 8.986 students registered in junior high and high schools (13–19 years)	88%	1.6% (*n* = 143) teenagers had been exposed to parental cancer; (6% of the parents had been diagnosed with cancer before the teenager had been born)	Age: fathers: M = 49 years, mothers: M = 45 years Gender: 49.2% female Main diagnoses: melanoma (17%), breast (15%), testicular (12%)	Age: 13–19 years Gender: 49.7% female
Joergensen et al. (36)	Denmark	Register-based data	Study population 1 (SP 1): *N* = 795.160 children 0–15 years born between 1986 and 1999; Study population 2 (SP 2): *N* = 360.054 children 0–18 years born between 1978 and 1984	NA^†^	4.3% (*n* = 34.373) children had been exposed to parental cancer (SP 1) 5.1% (*n* = 18.378) children had been exposed to parental cancer (SP 2)	Age: in 68.1% maternal age at child's birth was 25–34 years, in 57.2% paternal age at child's birth was 25–34 years Gender: 57.6% female	SP 1 ^§^: Age: 0–15 years; Gender: 50.1% female SP 2 ^§^: Age: 0–18 years Gender: 49.0% female
Martini et al. (37)	Australia	Register-based data and linkage with genealogy databases	*N* = 25.901 children affected by incident cancer diagnosis (1982–2015)	NA^†^	0.28% (*n* = 1.149) annual incidence of children being exposed to parental cancer in 2015	Age at diagnosis: M = 39.9 years Gender: 53% female Main diagnoses: breast, melanomas, digestive organs	Age: 0–11 years Gender: 48% female
Momen et al. (38)	Denmark	Register-based data	*N* = 2.158.430 children	NA^†^	0.13% (*n* = 2.725) of children being exposed to maternal cancer prenatal, 3.69% (*n* = 63.862) being exposed to maternal cancer postnatal	Age: maternal age in 46% ≥31years (prenatal exposure); maternal age in 48% 31years (postnatal exposure) Gender: 100% female Main diagnoses: NR	Age: 0–18 years Gender: 50% female (prenatal), 49% female (postnatal)
Morris et al. (39)	Australia	Register-based data and linkage with census data	*N* = 57.708 adolescents and young adults (12–24 years) affected by incident cancer diagnosis (1982–2015)	NA^†^	0.47% annual incidence of adolescents and young adults being exposed to parental cancer	Age at diagnosis: M = 51.3 Gender: 47.2% Female	Age at parent diagnosis: M = 18.8 Gender: 48.8% female
Niemelä et al. (40)	Finland	Finnish Birth Cohort Study and linkage with register-based data	*N* = 59.476 children	NA^†^	6.6% (*n* = 3.909) had been exposed to parental cancer at time of diagnosis 4.5% of the children were under the age of 18 years	Age: maternal age at child's birth M = 31.4 years Gender: 60.7% female Main diagnoses: NR^‡^	Age: 21 years at follow up Gender: 48.5% female
Syse et al. (43)	Norway	Register-based data	*n* =34.677 children (<18 years) among 20.843 cancer patients; *n* = 31.592 young adults (19–25 years) among 24.018 cancer patients	NA^†^	3.1% (*n* = 34.250) minor children had been exposed to parental cancer, 8.4% (*n* = 30.769) young adult children had been exposed to parental cancer, 0.3% annual incidence of families with minor children being exposed to parental cancer in 2007	see Table 1	Age: minors 0–18 years, young adults 19–25 years Gender: NR^‡^
Verkooijen et al. (41)	Sweden	Register-based data	*N* = 3.868.496 children born between 1960 and 2002	NA^†^	4.5% (*n* = 174.893) children had been exposed to maternal cancer;	Age: maternal age at child's birth between 16–46 years; Gender: 100% female Main diagnoses: breast, reproductive, digestive, melanoma	Age at diagnosis: 4.2% 1–18 years, 0.05% born ± 1 year, 0.2% not yet born Gender: NR^‡^

† *not applicable (register-based data);*

‡ *not reported;*

§ *study population*.

In a representative survey from Germany, 4.1% of the children (4–18 years) had a physically ill parent, about 1/3 of these had a parent with cancer (32). In a study including individuals with childhood psychiatric diagnosis (age at diagnosis <15 years), 2.2% of the children were exposed to a parental cancer diagnosis at the age between 0 and 15 years, whereas 6.8% had been exposed to parental cancer in their lifetime (33). Three articles based on different sub-populations of register-based data from Sweden reported that between 4.4 and 5.5% of the children (1–18 years) had been exposed to parental cancer (29, 34, 41). An article based on the Finnish Birth Cohort Study 1987 identified 4.5% of children (0–18 years) having a parent with cancer. Expanding the age range to 0–21 years, the prevalence was 6.6% (40). A publication from Norway reported that 3.1% of the children (0–18 years) had a parent with cancer (43). In a study with teenagers, 1.6% of the participating teenagers (13–19 years) had a parent with cancer (35). In young adults (19–25 years) a prevalence of 8.4% was reported (43). A publication based on a Danish sample found that 4.3% of children between 0 and 15 years and 5.1% of children between 0 and 18 years had been exposed to parental cancer (36). Another study from Denmark focusing on maternal cancer revealed that 2.7% of children between 0 and 18 years experienced a maternal cancer diagnosis (39).

Four articles presented estimate rates on children experiencing incident cancer diagnosis of a parent annually. Estimate rates ranged between 0.28% (0–11 year old children, Western Australia) (37), 0.30% (0–18 year old children, Norway) (43), 0.38% (0–18 year old children, Japan) (43), and 0.48% (12–24 year old children, Western Australia) (39) of children resp. young adults from the age-based total population experiencing a new cancer diagnosis in a parent within a 12 months period.

### Well-Being of Children Having a Parent With Cancer

Seven included population-based studies reported information on the psychosocial situation or well-being of children having a parent with cancer. These aspects were assessed using different instruments and assessment strategies, which results in different prevalence rates (Table 3). Documentation of psychiatric care use and documented psychiatric diagnosis yielded prevalence rates between 2.5 and 16.2% (38, 40, 42, 46) in different samples (e.g., regarding age, gender). One study including parental report of cancer survivors reported a prevalence rate of 10.8% of distress in children (6–18 years) up to 6 years after diagnosis parental cancer diagnosis (44). A publication on stress resilience in young adult men (~18 years) yielded low stress resilience in 21.4% of men with a parent with cancer (34). In a study including adolescents based on self-rated questionnaires on several psychosocial problems (e.g., self-esteem, eating problems), rates of several symptoms were between 6 (feeling lonely) and 41% (school problems). Summarizing the scales, 34% of adolescents yielded as high total problem cases (35).

**Table 3:** Study characteristics and estimates of psychosocial situation or well-being of children and/or young adults having a parent with cancer from *n* = 7 articles.

**Author, year**	**Database and data source**	**Sample**	**Characteristics of children: age; gender**	**Assessment of psychosocial situation or well-being**	**Information on well-being or psychosocial outcome in children having a parent with cancer**
Bultmann et al. (44)	Cancer registries, Parental report	*n* = 1,449 children of *n* = 976 cancer survivors	Age: M = 13.0 years (range 6-18 years); Gender: 47.9% female	Single item on distress (6-point-likert scale, cut off ≥3=distressed), Health related quality of life (Kidscreen-10 Index)	10.8% of the children were currently distressed; Overall: Higher HRQoL in children affected by parental cancer compared to norm population
Chen et al. (34)	Register-based data	*N* = 1.791.565 children (born between 1983 and 2000)	Age: 0–27 years Gender: 52.8% male	Diagnosis of psychiatric disorder (ICD-Code)	8.7% (male, cancer during pregnancy: 1%, female, cancer during pregnancy: 1.2%; male, cancer after birth: 7.6%, female, cancer: after birth 9.8%); higher risk of psychiatric disorder diagnoses in children of parents with cancer
Chen et al. (34)	Register-based data	*N* = 465.249 young men undergoing military conscription examination	Age: approximately 18 years (0–17 at time of parental diagnoses) Gender: 100% male	Semi-structured interview to assess ability to cope with psychological stress during military service (Stanine scale; score of 1–3=low stress resilience)	21.4% had low stress resilience; higher risk for low stress resilience in children of parents with cancer
Jeppesen et al. (35)	Representative sample and linkage with cancer registry	*N* = 8.986 students registered in junior high and high schools (13–19 years)	Age: 13–19 years Gender: 49.7% female	Several items/instruments assessing different aspects of psychosocial problems (somatic stress symptoms, cut off ≥ 2 symptoms; feeling lonely, 1 = very often/often; eating problems (EAT 7, cut off >4); self-esteem, 0–6 = low self-esteem; anxiety and depression (SCL-5, cut off ≥2), school problems, 1 = one or more problems; psychosocial problems, cut off ≥2);	10% eating problems, 6% feeling lonely, 20% low self-esteem, 18%anxiety/depression, 41% school problems, 36% somatic stress symptoms, high problem cases 34%; no differences between children of parents with cancer and control group
Momen et al. (38)	Register-based data	*N* = 2,725 children (prenatal exposure to cancer) *N* = 63.708 children (postnatal exposure to cancer)	Age: 0–18 years Gender: 50% female (prenatal exposure), 49% female (postnatal exposure)	Psychiatric diagnoses according to ICD-Code	3.4% (*n* = 93, prenatal exposure to cancer), 2.5% (*n* = 1,584, postnatal exposure to cancer)
Niemelä et al. (40)	Finnish Birth Cohort Study and linkage with register-based data	*N* = 59.476 children	Age: 21 years at follow up Gender: 48.5% female	Use of specialized psychiatric care	16.2% had received specialized psychiatric care
Niemelä et al. (16, 46)	Finnish Birth Cohort Study and linkage with register-based data	*N* = 59.476 children	Age: 21 years at follow up Gender: 48.5% female	Use of psychiatric outpatient care & psychiatric diagnoses according to ICD-Code	In total, 14% had received psychiatric outpatient care, 10.8% had been diagnosed with a mental disorder

*EAT, Eating Attitude Test; SCL, Symptom Check List*.

## Discussion

Cancer invades and causes ripple effects in the family. This narrative review compiles the prevalence rates of parental cancer from estimations of various population-based studies. Overall, the estimate rates varied among the populations due to different response rates or different characteristics of the included populations (e.g., age, gender). Eighteen publications met our inclusion criteria and of those, seven publications also reported information on the psychosocial situation or well-being of children having a parent with cancer.

The population-based study samples included between 8.144 and 151.756 cancer patients or cancer survivors and between 1.950 and 3.868.496 children. This review shows that cancer affects a considerable part of parents with between 14 and 24.7% of cancer patients having minor children, depending on age range of the sample.

In the future possibly even more children may experience parental cancer, since screenings programs may detect cancer earlier and more patients survive or live with cancer for many years due to treatment. Moreover, many people choose to have children later in life these days: in Germany about 27% of all women giving birth are older than 35 years (47). The mean age of having the first child in EU countries is about 29.1 years (48). Additionally, the transition to adulthood (leaving home) has shifted to later life: In the EU, men move from home at a mean age of 27 years and women at a mean age of 25 years (49). Taking this into account, makes it even more likely that, in future, cancer will be diagnosed more frequently among patients caring for minor and young adult children or living with young adult children in the same household.

The prevalence rates of children, adolescents and young adult children having a parent with a history of cancer range from 1.6 to 8.4% depending on the sample and age range taken into account. Studies on the well-being of children affected by parental cancer report estimate rates between 2.5 and 34% of children with substantial psychosocial burden. Compared to populations not being affected by parental cancer, the findings show mixed results. Some of the included articles found a slightly higher risk of a psychiatric diagnosis in children of parents with cancer compared to matched controls (34, 40, 45, 46), while other articles found no overall differences (35, 38). One article on quality of life found higher parent-reported quality of life in children of cancer survivors compared to norm values (44).

These findings indicate that children may not generally be at risk for psychopathological meaningful repercussions of the parental disease. As a matter of fact, studies in non-population-based samples of children affected by parental cancer identify higher distress and anxiety as well as lower quality of life compared to normative values (50, 51) and about one third of a sample of adolescents reported psychosocial problems (52). However, these samples were highly selective e.g., recruited among users of psychosocial support offers or in the context of cancer care for the parents.

Factors mediating the impact of parental cancer are e.g., family functioning or characteristics of the disease (11). Parents may provide adequate support to their children and families deal with a parental cancer diagnosis in an appropriate way, e.g., by communicating with their children openly or maintaining daily routines (53, 54). Moreover, families may have resources, which they use to cope with the situation and encounter the challenges of the parental disease (4, 55, 56).

While few publications in this review included questionnaires on psychosocial problems or stress based on proxy reports, most studies on estimate rates of psychological consequences do refer to psychiatric diagnoses based on data from health registries using DSM or ICD criteria. However, psychopathology might not be an appropriate indicator to identify psychosocial problems or burden of children facing parental cancer. Children may rather experience cancer specific concerns and fears, which may not be captured by general psychopathology since they might cause substantial mental burden, but do not meet the diagnostic criteria of psychiatric diseases (11, 57). Qualitative studies on children of cancer patients and studies using selected samples and more general instruments to detect stress symptoms or quality of life, indicate that children are experiencing substantial emotional burden, and hence, can benefit from supportive counseling (11, 14). Interventions to support children with a parent with cancer have been developed and shown to be valuable and helpful for children to cope with the potentially life-threatening disease of their parent and to deal with their own emotions (17, 58). Still, in current health care, minor children are not routinely included in health care and cooperation with child mental health care providers, as a preventive support offer, is rare (16).

Our findings on prevalence rates on affected parents and children underline the importance to routinely (1) assess the familial background of a patient and circulate this information within the health-care team and (2) assess the psychosocial situation of minor and young adult children as relatives of the patients. Our results reveal the necessity of an assessment of the psychosocial situation not only during acute treatment phase but also in aftercare. Where needed, psychosocial care for affected parents and children should be offered to all family members (18, 19).

### Limitations

Looking at the results of this review, some limitations need to be considered. Our primary interest was to review publications conducted in population-based studies. However, the results must be interpreted with caution since we included heterogeneous studies with different approaches and samples to estimate prevalence rates and used different criteria to assess mental health outcomes. The quality of included studies differs with regard to representativeness which e.g., might be caused by different sampling strategies and methodology. Indeed, the inclusion of population-based study reduces potential selection bias. But while some studies referred to patient and family registries with nearly complete data, other studies might have suffered from a selection bias due to incomplete response rates. All included studies were carried out in industrial countries and we cannot draw any conclusions with regard to developing countries. The strong difference in study populations hampers the possibility to provide an overall mean estimate for prevalence rates. Still, register-based data and some studies including nearly complete data allow for more precise estimates on prevalence rates than studies based on selected samples.

Results on the psychosocial situation and well-being of children affected by parental cancer should be interpreted with caution since not all studies included a control or comparison group from non-cancer samples. Moreover, cancer-specific burden may not be covered by the methods used in the included studies. Comparisons with non-population-based samples on children affected by parental cancer could not be conducted systematically due to incoherent use of assessment instruments across studies.

## Conclusions

Taking into account the substantial prevalence rates of cancer patients with minor children and the increasing survival rates of cancer patients during the past decades, it is important (1) to assess the familial background of the patients and (2) to support families in living with cancer and its consequences, e.g., fatigue, fears during after-care checkups, or limited capacity due to long-term side effects. Our review obtains an overview on prevalence data of cancer patients parenting minor or young adult children and children having a parent with cancer and, hence, help to determine the need for specific support services and health care planning. As between 14 and 22% of the cancer populations in this review were parents of minor and young adult children, expertise in disease coping and developmental aspects for children should be routinely integrated in psychosocial and psycho-oncological care teams.

Our findings suggest that children are not generally at risk for negative mental long-term consequences and that children may adjust well to parental cancer. Still, instruments to assess disease specific burden in children are lacking. Future studies should develop or refer to existing cancer-specific assessment tools. Moreover, the use of broadly used instruments on health-related quality of life or psychosocial problems may allow for comparisons with norm values as well as other disease groups.

Since every age group of children experiences different developmental aspects and tasks, a more distinct consideration of child age in future studies would be useful to provide a better understanding of needs during childhood. Longitudinal research including stage of illness, developmental stage of the children, and cancer-specific instruments to assess distress and needs are important and beneficial to investigate and understand the impact of parental cancer on parents as well as on children more thoroughly.

## Data Availability Statement

The original contributions presented in the study are included in the article/Supplementary Material, further inquiries can be directed to the corresponding author/s.

## Author Contributions

LI, LJ, and JB were involved in the data acquisition, data analysis, interpretation, and manuscript preparation. All authors were involved in the conception of the study, editing, contributed substantially to this manuscript, and reviewed the final version of this manuscript.

## Funding

This work was supported by German Cancer Aid (grant # 108303).

## Conflict of Interest

The authors declare that the research was conducted in the absence of any commercial or financial relationships that could be construed as a potential conflict of interest.

## Publisher's Note

All claims expressed in this article are solely those of the authors and do not necessarily represent those of their affiliated organizations, or those of the publisher, the editors and the reviewers. Any product that may be evaluated in this article, or claim that may be made by its manufacturer, is not guaranteed or endorsed by the publisher.

## References

[1] Visser A Huizinga GA van der Graaf WTA Hoekstra HJ Hoekstra-Weebers JEHM. The impact of parental cancer on children and the family: a review of the literature. Cancer Treat Rev. (2004) 30:683–94. 10.1016/j.ctrv.2004.06.001 15541578

[2] Edwards B Clarke V. The psychological impact of a cancer diagnosis on families: the influence of family functioning and patients' illness characteristics on depression and anxiety. Psychooncology. (2004) 13:562–76. 10.1002/pon.773 15295777

[3] Hyde MK Legg M Occhipinti S Lepore SJ Ugalde A Zajdlewicz L . Predictors of long-term distress in female partners of men diagnosed with prostate cancer. Psycho-Oncol. (2018) 27:946–54. 10.1002/pon.4617 29268006

[4] Semple CJ McCance T. Experience of parents with head and neck cancer who are caring for young children. J Adv Nurs. (2010) 66:1280–90. 10.1111/j.1365-2648.2010.05311.x 20546362

[5] Rauch PK Muriel AC. The importance of parenting concerns among patients with cancer. Crit Rev Oncl Hematol. (2004) 49:37–42. 10.1016/S1040-8428(03)00095-7 14734153

[6] Davey MP Bilkins B Diamond G Willis AI Mitchell EP Davey A . African American patients' psychosocial support needs and barriers to treatment: patient needs assessment. J Cancer Educ. (2016) 31:481–7. 10.1007/s13187-015-0861-9 26048632 PMC4671828

[7] Faulkner RA Davey M. Childr and adolescents of cancer patients: the impact of cancer on the family. Am J Fam Ther. (2002) 30:63–72. 10.1080/019261802753455651

[8] Gazendam-Donofrio SM Hoekstra HJ van der Graaf WTA van de Wiel HBM Visser A Huizinga G . Adolescents' emotional reactions to parental cancer: effect on emotional and behavioral problems. J Pediatr Psychol. (2011) 36:346–59. 10.1093/jpepsy/jsq090 20929959

[9] Visser A Huizinga GA Hoekstra HJ van der Graad WTA Gazedam-Donofrio SM Hoekstra-Weebers JE. Emotional and behavioral problems in children of parents recently diagnosed with cancer: a longitudinal study. Acta Oncol. (2007) 46:67–76. 10.1080/02841860600949560 17438707

[10] Thastum M Watson M Kienbacher C Piha J Steck B Zachariae R . Prevalence and predictors of emotional and behavioural functioning of children where a parent has cancer: a multinational study. Cancer. (2009) 115:4030–9. 10.1002/cncr.24449 19517480

[11] Morris JN Martini A Preen D. The well-being of children impacted by a parent with cancer: an integrative review. Support Care Cancer. (2016) 24:3235–51. 10.1007/s00520-016-3259-2 27079580

[12] Phillips F Lewis FM. The adolescent's experience when a parent has advanced cancer: a qualitative inquiry. Palliat Med. (2015) 29:851–8. 10.1177/0269216315578989 25855631

[13] Kennedy VL Lloyd-Williams M. How children cope when a parent has advanced cancer. Psycho-Oncol. (2009) 18:886–92. 10.1002/pon.1455 19137509

[14] Ellis SJ Wakefield CE Antill G Burns M Patterson P. Supporting children facing a parent's cancer diagnosis: a systematic review of children's psychosocial needs and existing interventions. Eur J Cancer Care. (2017) 26:e12432. 10.1111/ecc.12432 26776913

[15] Phillips F Prezio EA. Wonders & worries: evaluation of a child centered psychosocial intervention for families who have a parent/primary caregiver with cancer. Psycho-Oncol. (2017) 26:1006–12. 10.1002/pon.4120 26954773

[16] Niemelä M Marshall CA Kroll T Curran M Silverberg Koerner S Räsänen S . Family-focused preventive interventions with cancer cosurvivors: a call to action. Am J Public Health. (2016) 106:1381–7. 10.2105/AJPH.2016.303178 27196647 PMC4940637

[17] Niemelä M Hakko H Räsänen S. A systematic narrative review of the studies on structured child-centred interventions for families with a parent with cancer. Psychooncology. (2010) 19:451–61. 10.1002/pon.1620 19673009

[18] National Breast Cancer Centre and National Cancer Control Initiative. Clinical Practice Guidelines for the Psychosocial Care of Adults with Cancer. Camperdown, NSW: National Breast Cancer Centre (2003).

[19] National Compehensive Cancer Network. NCCN Guidelines for Patients:® Distress Management (2017). Retrieved from https://www.nccn.org/guidelines/patients

[20] Möller B Barkmann C Krattenmacher T Kühne F Bergelt C Beierlein V . Children of cancer patients: prevalence and predictors of emotional and behavioral problems. Cancer. (2014) 120:2361–70. 10.1002/cncr.28644 24957877

[21] Huizinga GA van der Graaf WTA Visser A Dijkstra JS Hoekstra-Weebers JEHM. Psychosocial consequences for children of a parent with cancer: a pilot study. Cancer Nurs. (2003) 26:195–202. 10.1097/00002820-200306000-00004 12832952

[22] Krattenmacher T Kühne F Halverscheid S Wiegand-Grefe S Bergelt C Romer G . A comparison of the emotional and behavioral problems of children of patients with cancer or a mental disorder and their association with parental quality of life. J Psychosom Res. (2004) 73:213–20. 10.1016/j.jpsychores.2013.11.020 24529040

[23] Visser A Huizinga GA Hoekstra HJ van der Graaf WTA Klip E Pras E . Emotional and behavioural functioning of children of a parent diagnosed with cancer: a cross-informant perspective. Psychooncology. (2005) 14:746–58. 10.1002/pon.902 15744787

[24] Moher D Liberati A Tetzlaff J Altmann DG. Preferred reporting items for systematic reviews and meta-analyses: the PRISMA statement. Ann Intern Med. (2009) 15:264–9. 10.7326/0003-4819-151-4-200908180-00135 19622511

[25] Wells GA Shea B O'Connell D Peterseon J Welch B Losos M . The Newcastle-Ottawa Scale (NOS) for Assessing the Quality of Non Randomised Studies in Meta-Analyses (2001). Available online at: http://www.ohri.ca/programs/clinical_epidemiology/oxford.asp (accessed April 25, 2019).

[26] Inhestern L Bultmann JC Beierlein V Möller B Romer G Koch U . Understanding parenting concerns in cancer survivors with minor and young-adult children. J Psychosom Res. (2016) 87:1–6. 10.1016/j.jpsychores.2016.05.008 27411745

[27] Inhestern L Bultmann JC Beierlein V Möller B Romer G Muriel AC . Psychometric properties of the parenting concerns questionnaire in cancer survivors with minor and young adult children. Psycho-Oncol. (2016) 25:1092–8. 10.1002/pon.4049 26677091

[28] Chen R Regodon Wallin A Sjölander A Valdimarsdóttir U Ye W Tiemeier H . Childhood injury after a parental cancer diagnosis. eLife. (2015) 4:31. 10.7554/eLife.08500 26519735 PMC4749389

[29] Chen R Sjölander A Valdimarsdóttir U Varnum C Almquist C Ye W . Parental cancer diagnosis and child mortality - a population-based cohort study in Sweden. Cancer Epidemiol. (2015) 39:79–85. 10.1016/j.canep.2014.11.011 25548077

[30] Ernst JC Beierlein V Romer G Möller B Koch U Bergelt C. Use and need for psychosocial support in cancer patients: a population-based sample of patients with minor children. Cancer. (2013) 119:2333–41. 10.1002/cncr.28021 23575997

[31] Weaver KE Rowland JH Alfano CM McNeel TS. Parental cancer and the family: a population-based estimate of the number of US cancer survivors residing with their minor children. Cancer. (2010) 116:4395–401. 10.1002/cncr.25368 20586037 PMC3164357

[32] Barkmann C Romer G Watson M Schulte-Markwort M. Parental physical illness as a risk for psychosocial maladjustment in children and adolescents: epidemiological findings from a national survey in Germany. Psychosomatics. (2007) 48:476–81. 10.1176/appi.psy.48.6.476 18071093

[33] Benros ME Laursen TM Dalton SO Nordentoft M Mortensen PB. The risk of schizophrenia and child psychiatric disorders in offspring of mothers with lung cancer and other types of cancer: a Danish nationwide register study. PLoS ONE. (2013) 8:e79031. 10.1371/journal.pone.0079031 24223877 PMC3815227

[34] Chen R Fall K Czene K Kennedy B Valdimarsdóttir U Fang F. Impact of parental cancer on IQ, stress resilience, and physical fitness in young men. Clin Epidemiol. (2018) 10:593–604. 10.2147/CLEP.S152210 29872348 PMC5973433

[35] Jeppesen E Bjelland I Fossa SD Loge JH Dahl AA. Psychosocial problems of teenagers who have a parent with cancer: a population-based case-control study (Young-HUNT Study). J Clin Oncol. (2013) 31:4099–104. 10.1200/JCO.2013.50.7061 24101041

[36] Joergensen AC Kjaer Urhoj S Nybo Andersen AM. Primary school achievement and socioeconomic attainment in individuals affected by parental cancer in childhood or adolescence: a Danish nationwide register-based study. J Epidemiol Community Health. (2018) 72:982–9. 10.1136/jech-2018-210472 30126977

[37] Martini A Morris JN Jackson HM Ohan JL. The impact of parental cancer on preadolescent children (0–11 years) in Western Australia: a longitudinal population study. Support Care Cancer. (2019) 27:1229–36. 10.1007/s00520-018-4480-y 30259135

[38] Momen NC Ernst A Arendt LH Olsen J Li J Gissler M . Mental and behavioural disorders in the children of mothers diagnosed with cancer: a Danish population-based register study. Psycho-Oncol. (2019) 28:408–14. 10.1002/pon.4958 30511799

[39] Morris JN Zajac I Turnbull D Preen S Patterson P Martini A. A longitudinal investigation of Western Australian families impacted by parental cancer with adolescents and young adult offspring. Aust N Z J Public Health. (2019) 43:261–6. 10.1111/1753-6405.12885 30830710

[40] Niemelä M Paananen R Hakko H Merikukka M Gissler M Räsänen S. The prevalence of children affected by parental cancer and their use of specialized psychiatric services: the 1987 Finnish Birth Cohort study. Int J Cancer. (2012) 131:2117–25. 10.1002/ijc.27466 22307957

[41] Verkooijen HM Ang JX Liu J Czene K Salim A Hartmann M. Mortality among offspring of women diagnosed with cancer: a population-based cohort study. Int J Cancer. (2013) 132:2432–8. 10.1002/ijc.27899 23047289

[42] Inoue I Higashi T Iwamoto M Heiney SP Tamaki T Osawa K . A national profile of the impact of parental cancer on their children in Japan. Cancer Epidemiol. (2015) 39 838–41. 10.1016/j.canep.2015.10.005 26651443

[43] Syse A Aas GB Loge JH. Children and young adults with parents with cancer: a population-based study. Clinical Epidemiol. (2012) 4:41–52. 10.2147/CLEP.S28984 22442635 PMC3307636

[44] Bultmann JC Beierlein V Romer G Möller B Koch U Bergelt C. Parental cancer: health-related quality of life and current psychosocial support needs of cancer survivors and their children. Int J Cancer. (2014) 135:2668–77. 10.1002/ijc.28905 24740862

[45] Chen R Regodon Wallin A Selinus EN Sjölander A Fall K Valdimarsdóttir U . Psychiatric disorders among children of parents with cancer: a Swedish register-based matched cohort study. Psycho-Oncol. (2018) 27:1854–60. 10.1002/pon.4738 29663601

[46] Niemelä M Paananen R Hakko H Merikukka M Gissler M Räsänen S. Mental disorder diagnoses of offspring affected by parental cancer before early adulthood: the 1987 Finnish Birth Cohort study. Psycho-Oncol. (2016) 25:1477–84. 10.1002/pon.4088 26857036

[47] Statistisches Bundesamt. Anzahl der Geburten nach dem Alter der Mutter in Deutschland im Jahr 2017 (2018). Available online at: https://de.statista.com/statistik/daten/studie/161856/umfrage/geburten-nach-dem-alter-der-mutter-in-deutschland/ (accessed April 25, 2019).

[48] Eurostat. Statistical Office of the European Union Mean Age of Women at Childbirth and at Birth of First Child (2018). Available online at: https://ec.europa.eu/eurostat/web/products-datasets/-/tps00017 (accessed April 25, 2019).

[49] Eurostat. Statistical Office of the European Union Being Young in Europe Today - Family and Society. (2017). Available online at: https://ec.europa.eu/eurostat/statistics-explained/index.php/Being_young_in_Europe_today_-_family_and_society (accessed April 25, 2019).

[50] McDonald FEJ Patterson P White KJ Butow PN Costa DSJ Kerridge I. Correlates of unmet needs and psychological distress in adolescent and young adults who have a parent diagnosed with cancer. Psycho-Oncol. (2016) 25:447–54. 10.1002/pon.3942 26282864

[51] Hauken MA Senneseth M Dyregrov A Dyregrov K. Anxiety and the quality of life of children living with parental cancer. Cancer Nurs. (2018) 41:E19–27. 10.1097/NCC.0000000000000467 28085693

[52] Krattenmacher T Kühne F Führer D Beierlein V Brähler E Resch F . Coping skills and mental health status in adolescents when a parent has cancer: a multicenter and multi-perspective study. J Psychosom Res. (2013) 74:252–9. 10.1016/j.jpsychores.2012.10.003 23438718

[53] Buchbinder M Longhofer J McCue K. Family routines and rituals when a parent has cancer. Fam Sys Health. (2009) 27:213–27. 10.1037/a0017005 19803616

[54] Davey MP Nino A Kissil K Ingram M. African American parents' experiences navigating breast cancer while caring for their children. Qual Health Res. (2012) 22:1260–70. 10.1177/1049732312449211 22767699

[55] Inhestern L Bergelt C. When a mother has cancer: strains and resources of affected families from the mother's and father's perspective-a qualitative study. BMC Womens Health. (2018) 18:72. 10.1186/s12905-018-0562-8 29801481 PMC5970502

[56] Kissil K Nino A Ingram M Davey M. “I knew from day one that I'm either gonna fight this thing or be defeated”: African American parents' experiences of coping with breast cancer. J Fam Nurs. (2014) 20:98–119. 10.1177/1074840713504035 24027088

[57] Patterson P McDonald FEJ Butow P White KJ Costa DSJ . Psychometric evaluation of the Offspring Cancer Needs Instrument (OCNI): an instrument to assess the psychosocial unmet needs of young people who have a parent with cancer. Support Care Cancer. (2013) 21:1927–38. 10.1007/s00520-013-1749-z 23420556

[58] Kühne F Krattenmacher T Beierlein V Grimm JC Bergelt C Romer G . Minor children of palliative patients: a systematic review of psychosocial family interventions. J Palliat Med. (2012) 15:931–45. 10.1089/jpm.2011.0380 22849598 PMC3396138

