# Supplementary material for: Estimates of Prevalence Rates of Cancer Patients With Children and Well-Being in Affected Children: A Systematic Review on Population-Based Findings
Source: Front Psychiatry. 2021 Nov 25;12:765314. doi: 10.3389/fpsyt.2021.765314 (PMC8656299; doi:10.3389/fpsyt.2021.765314)
Supplement: Supplementary file 1 [file Table_1.docx]

Supplemental Table 1: *Inclusion and exclusion criteria*

| Inclusion criteria | |
| --- | --- |
| (1) | Accessibility of full text |
| (2) | Language English or German |
| (3) | Article published in a peer-reviewed journal |
| (4) | Study population: cancer patients with minor children/ minor children of cancer patients |
| (5) | Based on population-based data |
| (6) | Report of population-based estimates and characteristics of prevalences of cancer patients with children and young adults (≤ 25 years) and/or children or young adults (≤ 25 years) affected by parental cancer |
| Exclusion criteria | |
| (1) | Other language than English or German |
| (2) | Article published in a journal without peer-review |
| (3) | Study population: cancer patients with children > 25 years of cancer patients |
| (4) | No original research |
| (5) | Only qualitative methodology used |
| (6) | No population-based estimated reported explicitly for parental cancer but parental illness in general |
