# Supplementary material for: Estimates of Prevalence Rates of Cancer Patients With Children and Well-Being in Affected Children: A Systematic Review on Population-Based Findings
Source: Front Psychiatry. 2021 Nov 25;12:765314. doi: 10.3389/fpsyt.2021.765314 (PMC8656299; doi:10.3389/fpsyt.2021.765314)
Supplement: Supplementary file 2 [file Table_2.docx]

Supplemental Table 2. Quality assessment of the included articles (n=18) using modified criteria adapted from the Newcastle Ottawa Scale

|  | Selection | |  |  | Outcome  (maladjustment/distress in children of parents with cancer) | | Total number of “yes” |
| --- | --- | --- | --- | --- | --- | --- | --- |
| Author (years) | Is the sample truly or somewhat representative of the average of the target population? | Is the response rate satisfactory? | Is the sample size satisfactory? | Is the ascertainment of exposure based on secure records or structured self-report? | Is the assessment of the outcome valid and reliable? | Is the statistical tests to analyze the data appropriate? |  |
| Barkmann et al., 2007 | yes | no | yes | yes | n/a | n/a | 3/4 |
| Benros et al., 2013 | yes | yes | yes | yes | n/a | n/a | 4/4 |
| Bultmann et al., 2014 | unclear | no | yes | yes | yes | yes | 4/6 |
| Chen et al., 2015 | yes | yes | yes | yes | n/a | n/a | 4/4 |
| Chen et al., 2015 | yes | yes | yes | yes | n/a | n/a | 4/4 |
| Chen et al., 2018a | yes | yes | yes | yes | yes | yes | 6/6 |
| Chen et al., 2018b | yes | yes | yes | yes | yes | yes | 6/6 |
| Ernst et al., 2013 | unclear | no | yes | yes | n/a | n/a | 2/4 |
| Inoue et al., 2015 | yes | yes | yes | yes | n/a | n/a | 4/4 |
| Jeppesen et al., 2013 | unclear | yes | yes | yes | unclear | yes | 4/6 |
| Joergensen et al., 2018 | yes | yes | yes | yes | n/a | n/a | 4/4 |
| Martini et al., 2019 | yes | yes | yes | yes | n/a | n/a | 4/4 |
| Momen et al., 2018 | yes | yes | yes | yes | yes | yes | 6/6 |
| Morris et al., 2019 | yes | yes | yes | yes | n/a | n/a | 4/4 |
| Niemelä et al., 2012 | yes | yes | yes | yes | yes | yes | 6/6 |
| Niemelä et al., 2016 | yes | yes | yes | yes | yes | Yes | 6/6 |
| Syse et al. (2012) | yes | yes | yes | yes | n/a | n/a | 4/4 |
| Verkooijen et al., 2013 | yes | yes | yes | yes | n/a | n/a | 4/4 |
| Weaver et al. (2010) | unclear | no | yes | yes | n/a | n/a | 2/4 |

n/a, not applicable
